# Functional Connectome Controllability in Patients with Mild Cognitive Impairment after Repetitive Transcranial Magnetic Stimulation of the Dorsolateral Prefrontal Cortex

**DOI:** 10.3390/jcm13185367

**Published:** 2024-09-10

**Authors:** Simone Papallo, Federica Di Nardo, Mattia Siciliano, Sabrina Esposito, Fabrizio Canale, Giovanni Cirillo, Mario Cirillo, Francesca Trojsi, Fabrizio Esposito

**Affiliations:** 1 Department of Advanced Medical and Surgical Sciences (DAMSS), University of Campania “Luigi Vanvitelli”, 80138 Naples, Italy; simone.papallo@unicampania.it (S.P.); federica.dinardo@unicampania.it (F.D.N.); mattia.siciliano@unicampania.it (M.S.); fabrizio.canale@unicampania.it (F.C.); mario.cirillo@unicampania.it (M.C.); fabrizio.esposito@unicampania.it (F.E.); 2 First Division of Neurology and Neurophysiopathology, University Hospital, University of Campania “Luigi Vanvitelli”, 80138 Naples, Italy; sabrina.esposito1@unicampania.it; 3 Department of Mental and Physical Health and Preventive Medicine, University of Campania “Luigi Vanvitelli”, 80138 Naples, Italy; giovanni.cirillo@unicampania.it

**Keywords:** mild cognitive impairment, repetitive TMS, resting state functional MRI, functional connectome, network control theory, controllability

## Abstract

**Background**: Repetitive transcranial magnetic stimulation (rTMS) has shown therapeutic effects in neurological patients by inducing neural plasticity. In this pilot study, we analyzed the modifying effects of high-frequency (HF-)rTMS applied to the dorsolateral prefrontal cortex (DLPFC) of patients with mild cognitive impairment (MCI) using an advanced approach of functional connectome analysis based on network control theory (NCT). **Methods**: Using local-to-global functional parcellation, average and modal controllability (AC/MC) were estimated for DLPFC nodes of prefrontal-lateral control networks (R/LH_Cont_PFCl_3/4) from a resting-state fMRI series acquired at three time points (T0 = baseline, T1 = T0 + 4 weeks, T2 = T1 + 20 weeks) in MCI patients receiving regular daily sessions of 10 Hz HF-rTMS (*n* = 10, 68.00 ± 8.16 y, 4 males) or sham (*n* = 10, 63.80 ± 9.95 y, 5 males) stimulation, between T0 and T1. Longitudinal (group) effects on AC/MC were assessed with non-parametric statistics. Spearman correlations (ρ) of AC/MC vs. neuropsychological (RBANS) score %change (at T1, T2 vs. T0) were calculated. **Results:** AC median was reduced in MCI-rTMS, compared to the control group, for RH_Cont_PFCl_3/4 at T1 and T2 (vs. T0). In MCI-rTMS patients, for RH_Cont_PFCl_3, AC % change at T1 (vs. T0) was negatively correlated with semantic fluency (ρ = −0.7939, *p* = 0.045) and MC % change at T2 (vs. T0) was positively correlated with story memory (ρ = 0.7416, *p* = 0.045). **Conclusions**: HF-rTMS stimulation of DLFC nodes significantly affects the controllability of the functional connectome in MCI patients. Emerging correlations between AC/MC controllability and cognitive performance changes, immediately (T1 vs. T0) and six months (T2 vs. T0) after treatment, suggest NCT could help explain the HF-rTMS impact on prefrontal-lateral control network, monitoring induced neural plasticity effects in MCI patients.

## 1. Introduction

Mild cognitive impairment (MCI) is a neurological condition characterized by a cognitive decline greater than expected (in relation to a patient’s age and education) affecting different domains, including memory, language, attention, visuospatial, and executive functions [1,2,3] and is often used to describe an intermediate stage of cognitive decline between healthy aging and dementia [4]. However, MCI is usually not a stable condition over time and may often exhibit a variable probability of progressing toward worse cognitive impairment [5]. Indeed, MCI can be viewed as the prodromal stage of a variety of dementing disorders, including Alzheimer’s disease (AD), for which different studies reported a yearly incidence of 6~25% of MCI patients likely to convert to AD [6,7,8,9]. Over the last decade, several therapeutic strategies were considered to possibly delay or prevent the progression from MCI to AD [5,10,11,12]. However, conventional (drug) therapies are often limited to symptoms and burdened by long-term undesirable (side) effects [13], whereas, new research on therapies based on non-invasive brain stimulation has produced some promising results [13]. In particular, several works have assessed the impact of the high frequency repetitive transcranial magnetic stimulation (HF-rTMS) [14,15], reporting changes in the cortical excitability [14,16] as well as in the functional connectivity of brain large-scale networks and in the cognitive performances [17], suggesting that this kind of neurostimulation might provide a viable and sustainable treatment option for MCI. More specifically, in our recent work (Esposito et al., 2022) [17], the short- (immediately after a 4-week treatment) and long-term (after 6 months from the end of treatment) effects of an HF-rTMS of the dorsolateral prefrontal cortex (DLPFC) has been described on the brain large-scale functional connectivity. In particular, we reported both short- and long-term significant increases in the functional connectivity in some brain regions crucial for executive function. Moreover, we evaluated the cognitive performances via the repeatable battery for the assessment of neuropsychological status (RBANS) [18], reporting significantly better performances in semantic fluency and orientation line scores immediately after the 4-week treatment.

In light of previous results, here we considered an alternative approach to account for the modifying effects of the stimulation of the so-called functional connectome [19]. Namely, here we applied network control theory (NCT) modeling, a relatively newer theoretical framework for the study of human brain functional connectivity, to more specifically address the possible causal influence of individual cortical sites on the entire connectome in terms of their general ability to efficiently induce (or facilitate) significant changes in the state of the network [20].

NCT has been previously introduced with the express purpose of characterizing the ability to steer, e.g., via brain stimulation, the state of a network towards a target state by driving certain “input” nodes [21]. In this way, NCT has allowed the creation of a unifying account of local influences on global brain dynamics, enabling a more effective (and possibly even predictive) modeling of the intrinsic mechanism of causality underlying the neuromodulatory effects of a (hypothetical) neurostimulation at given sites. Although the majority of previous NCT applications have been based on structural connectivity network data, as obtained via diffusion-weighted MRI, the change in the information flow through the connectome is what would cause the brain to switch between (or encompass several) different functional states. Thereby, because the brain functional state can be characterized for a given period of observation from the joint levels of functional (co-)activation at all connectome nodes, in this pilot study, we extracted the two most typical NCT metrics from regional fMRI time series, attempting to explain both shorter and longer lasting perturbations in this mechanism, after HF-rTMS, in a small group of MCI patients.

An NCT analysis allows estimating two main local metrics of “controllability” for the human connectome at each single node of a given parcellation of the brain. Here, “controllability” refers to the property of a given node to be a “good place” from where the connectome would be driven (e.g., via neurostimulation) to more efficiently attain (or maintain over time) a given functional state [19,22,23]. In particular, the so-called average controllability (AC) and modal controllability (MC) quantify the ability of the system to be driven via a single node, respectively, towards relatively more “consolidated” states and relatively more “difficult-to-reach” states [24]. A consolidated state would be a state that is well-established from previous recurrent experiences and therefore expected to occur naturally and frequently during a given period of observation. In contrast, a non-consolidated or difficult-to-reach state would be one that can be reached under more cognitively engaged conditions (e.g., via certain task demands and/or during effortful cognitive stimulation) and therefore expected to occur less frequently during a given period of “unconstrained” observation, such as, e.g., when the subject is at rest. 

As an essential part of the executive large-scale network, the DLPFC is a region well known to be involved in the monitoring of information in working memory (see, e.g., [25] for a review on the architectonic and functional organization of the whole lateral prefrontal cortex). As a consequence, it is often hypothesized (and sometimes hinted by experimental data) that the DLPFC would be also significantly implicated in the active driving of the entire functional connectome toward more difficult-to-reach functional states [26,27], in which particular case, the MC metric at this site would be a promisingly good indicator of the natural capability of the human connectome to effectively control the information flow, as is normally required when a subject is asked to perform a working-memory task [22,28]. Starting from this assumption, here we analyzed the effects of the HF-rTMS stimulation on the human functional connectome in terms of the estimated AC and MC levels at all nodes encompassing the DLPFC as well as the possible correlation between their changes and the changes in three RBANS scores (semantic fluency, line orientation, and story memory) already known to be useful to assess working memory functioning and related performances in high-order cognitive tasks in MCI individuals [29].

## 2. Materials and Methods

### 2.1. Study Design

All details about the rTMS protocol and the study cohort, including the criteria for recruitment, their clinical and neurological assessment, and the detailed procedures for MRI data acquisition and pre-processing can be found in the study performed by Esposito et al. (2022) [17].

Briefly, twenty MCI patients were extracted from the patients enrolled by Esposito et al. (2022) [17]. Because we aimed to merely test longitudinal effects using a fully balanced factorial data model, i.e., with no missing data points, we only extracted datasets and metrics from MCI patients who had successfully completed the entire treatment and assessment protocol. This longitudinal study included the MRI acquisition and the neuropsychological assessment at three time points: immediately before starting the stimulation sessions, at baseline (T0), immediately after the 4-week rTMS (or sham) treatment (T1 = T0 + 4 weeks), and after 6 months, i.e., 20 weeks after the end of the treatment (T2 = T1 + 20 weeks). In accordance with the double-blind experimental design of the study protocol presented by Esposito et al. (2022) [17], MCI patients were randomly assigned to either the active or sham (control) group. None of the patients had previous experience of rTMS and neither the patients, nor the researchers assessing their cognitive performances, knew whether the patients were receiving active or sham rTMS. Here, the same two groups were considered to separate patients who actually underwent a four-week rTMS treatment (MCI-TMS group: 10 subjects, 68.00 ± 8.16, 4 males) and patients who actually underwent identical sessions with a four-week sham treatment (MCI-C: 10, subjects, 63.80 ± 9.95, 5 males). The rTMS and sham sessions included a high-frequency (10 Hz) stimulation applied over the DLPFC for 10 min to the left (or right) side and for 10 min to the right (or left) side, the order of stimulation sites (left first or right first) being arbitrarily chosen in such a way to be balanced between the two groups. The magnetic stimulator, the operation process, and the sounds generated during treatment were the same for the MCI-TMS and MCI-C groups, the stimulation parameters being consistent between the active and sham sessions. However, for the sham condition, a placebo coil with a mechanical outline and sound level (click) identical to the active one was used, delivering less than 5% of the magnetic output to the patients. Each r-TMS or sham session was repeated 5 times per week (on separate weekdays) for 4 weeks.

Resting-state blood oxygen level-dependent (BOLD) fMRI images were registered to 3D T1-weighted MRI images, which were normalized to the MNI template to provide the transformations necessary to apply a normative brain parcellation to functional data in the native space. One hundred bilateral cortical nodes were derived from the functional local–global normative parcellation [30] and accordingly categorized into sets of nodes corresponding to the seven large-scale canonical functional networks. In particular, the executive control subnetwork of the human connectome atlas, i.e., the prefrontal control lateral (PFCl) network, encompassing all regions belonging to the DPLFC, i.e., the stimulation site, was considered [31]. The brain parcellation applied to the data resulted in a 100 (node/regions) × 320 (time points) functional time-course matrix (as obtained by averaging the BOLD fMRI time series across all voxels in the corresponding atlas region) per each subject.

Repeatable battery for the assessment of neuropsychological status (RBANS) forms A, B, and C [32] were used to assess the cognitive performances of the study groups at the three time points. From these forms, and according to the results already presented by Esposito et al. (2022), we focused on three neuropsychological scores, i.e., the story memory-IR, line orientation, and Semantic fluency, for which we had collected previous evidence of significantly reduced scores at T0 in both MCI groups compared to an independent group of sex- and age-matching healthy control subjects. 

### 2.2. Controllability Metrics

**Figure 1:**
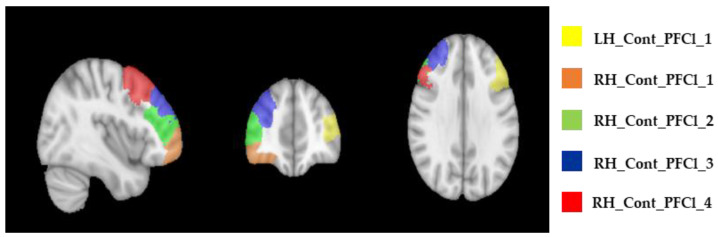
Localization of the stimulation site. Resorting to the 100-region Schaefer parcellation, we identified the functional connectome nodes encompassing the nominal stimulation site (DLPFC). The LH_Cont_PFCl_1 (Yellow), RH_Cont_PFCl_1 (Orange), RH_Cont_PFCl_2 (Green), RH_Cont_PFCl_3 (Blue), and RH_Cont_PFCl_4 (Red) are here reported in the sagittal, coronal, and transversal planes.

The functional connectivity matrix was estimated via Pearson correlation coefficients between the time series of each node. This resulted in three 100 × 100 symmetric connectivity matrices per participant for each time point (T0, T1, and T2). Starting from the basic NCT model formulation [33], we derived estimates for the average (AC) and modal (MC) controllability of five functional connectome regions encompassing the stimulation sites (see Figure 1), namely, of LH_Cont_PFCl_1, RH_Cont_PFCl_1, RH_Cont_PFCl_2, RH_Cont_PFCl_3, and RH_Cont_PFCl_4. Considering the minimum energy control principle to determine the evolution of the system through different activation states system and applying a plausible normalization to the connectivity matrices [34], the average and the modal controllability were estimated using the formulation proposed, and operationally adapting the Matlab code made shared by Deng et al. (2022) [33]. In particular, the average controllability (AC) equals the average input energy from a control node and over all possible target states [35]. Thus, the AC quantifies the capability of a given stimulated brain region (node), on average, to drive the system (i.e., the whole connectome) into so-called “easy-to-reach” states, i.e., states that are frequently and naturally occurring during a given period of observation of the neural time series when the subject is not requested to perform any task (e.g., resting state). In contrast, modal controllability (MC) refers to the eigenvalues of the normalized connectivity matrices according to the formulation proposed in the literature [19,35]. The MC quantifies the capability of a given stimulated brain region (node) to drive the system into so-called “difficult-to-reach” states, i.e., states that are not frequently occurring during a given period of observation of the neural time series with the subject in the resting state. These states would be achieved with substantially more input energy at the node as would be the case when the subject is more systematically engaged or attention-focused on executing a demanding cognitive task [19]. In particular, as DLPFC encompasses multiple nodes of the PFCl control network, MC estimates can be expected relatively higher (and correspondingly AC estimates relatively lower) for these nodes, as far as the role of this region in complex cognitive tasks is preserved, i.e., higher activity during higher-level cognitive task performances (e.g., involving working memory) and reduced activity during the resting state [22].

### 2.3. Statistical Analysis

At baseline (T0), we used the Mann–Whitney U test (MWU) and Pearson’s chi-squared test (χ^2^ test) to compare the two study groups on age and sex, respectively. First, a two-way analysis of variance (ANOVA) model, including one between-subject factor with two levels (MCI-TMS and MCI-C) and one within-subject factor with three levels (T0, T1, T2), was applied to assess any two-way interaction effects between the two factors. Here, MWU tests were performed post hoc to compare the controllability estimates between the two groups at each time point. Second, a one-factor repeated measures ANOVA was performed for each group separately to determine whether the AC and MC estimates varied significantly across the three time points. Here, Wilcoxon signed-rank tests for pairwise post hoc comparisons were performed. Finally, at T1 and T2, we also compared the AC and MC of the two study groups by using an analysis of covariance (ANCOVA) model to consider the AC and MC estimates at T0 as covariates. All post hoc comparisons were performed with non-parametric statistical tests due to the small number of subjects per group, but the resulting *p*-values were only considered significant after successful correction for multiple comparisons using the Benjamini–Hochberg procedure [36] to estimate the false discovery rate (FDR). More specifically, *p*-values resulting in corresponding FDR ≤ 0.05 were considered statistically significant.

The Spearman correlation (ρ) between the variation, i.e., the percent change at T1 or T2 with respect to T0, of AC or MC and three cognitive scores, namely story memory-IR, line orientation, and semantic fluency, were calculated.

All statistical analyses were performed Matlab R2022b (The Mathworks, Inc., Natick, MA, USA, www.mathworks.com) and RStudio 4.2.3 (Integrated Development Environment for R. Posit Software, PBC, Boston, MA, http://www.posit.co/).

## 3. Results

The two groups (MCI-TMS, MCI-C) did not differ significantly in age (MWU test, *p* > 0.05) and in sex (χ^2^ = 0.2198, *p* > 0.05) between MCI-TMS and MCI-C groups. At baseline (T0), the two groups did not differ significantly in any of the cognitive scores (Table 1).

**Figure 2:**
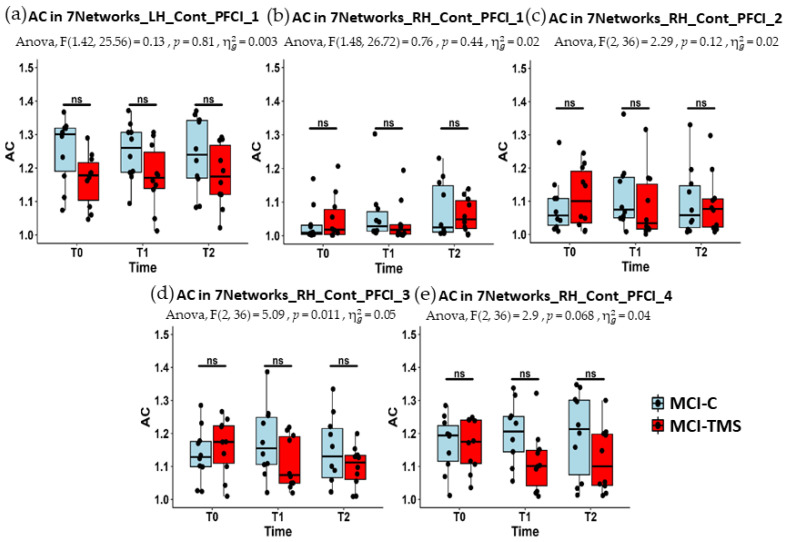
The boxplots of AC estimates for both groups (in light blue, MCI-C; in red, MCI-TMS; the black points for the AC estimates of each subject) across time points (T0, T1, and T2) for the five regions, namely, LH_Cont_PFCl_1 (**a**), RH_Cont_PFCl_1 (**b**), RH_Cont_PFCl_2 (**c**), RH_Cont_PFCl_3 (**d**), and RH_Cont_PFCl_4 (**e**). The two-way ANOVA with one within factor (observation time) and one between factors (group membership) showed that the AC was significantly affected by the interaction term, i.e., the interplay between the within and between factors, in RH_Cont_PFCl_3 (F (2, 36) = 5.09, *p* = 0.01), as reported in the subtitle of the corresponding plot. The Mann–Whitney test (U) as a post hoc test was performed, but no significant difference between the groups at each time point was obtained. In no other selected regions did the interaction term result as statistically significant. FDR correction for multiple comparisons was considered.

In Figure 2, we report the boxplots of the AC estimates of the selected functional brain regions for both MCI-C and MCI-TMS at each time point (T0, T1, and T2). In Table 2 and Table 3, we report the median and inter-quartile range (IQR) of the AC estimates of the five selected regions across the three time points for the MCI-C and MCI-TMS groups, respectively. The two-way ANOVA showed a statistically significant two-way interaction in the AC estimates in RH_Cont_PFCl_3 (F (2, 36) = 5.09, *p* = 0.01) but not in the other selected regions. However, post hoc MWU tests did not provide sufficient evidence for group differences at specific time points.

**Figure 3:**
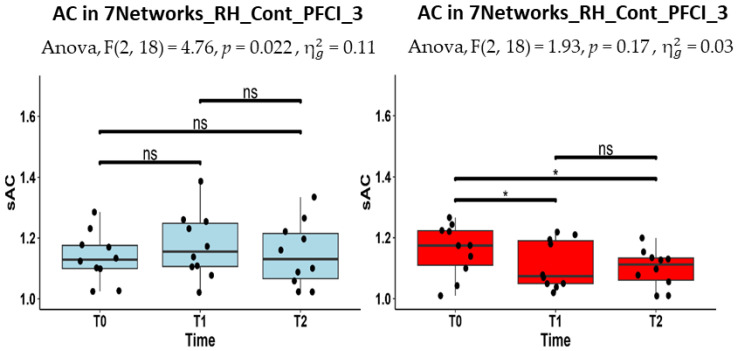
The boxplots of AC estimates (the black dots) across time points (T0, T1 and T2) for the MCI-C (in light blue) and for the MCI-TMS (in red) are here reported separately for the RH_Cont_PFCl_3, where the repeated measure ANOVA was significant, when considering the MCI-TMS group, as reported in the subtitle. The Wilcoxon signed-rank test was performed to evaluate short- and long-term changes of the AC estimates of the considered region. Short- and long-term statistically significant reduction (*p* < 0.05) for the AC estimates was found (after FDR correction, * *p* < 0.05) in the MCI-TMS group but not in MCI-C group.

In Figure 3, we report the boxplots of the AC estimates of this region for the two groups, separately. The one-factor repeated measure ANOVA on the AC estimates (i.e., with the two groups analyzed separately) revealed a statistically significant effect of time in the RH_Cont_PFCl_3 node (F (1,17) = 4.76, *p* = 0.022) in the MCI-TMS group but not in the MCI-C group. The Wilcoxon signed-rank tests for the pairwise post hoc comparisons showed that, only in the MCI-TMS group, the AC values significantly decreased at T1 (*p* = 0.043) and at T2 (*p* = 0.043) with respect to the baseline (at T0). Moreover, the one-factor ANCOVA model (i.e., with T1 and T2 estimates analyzed separately with T0 estimates as covariates) revealed that the AC estimates at T1 significantly differed between the two groups in the nodes RH_Cont_PFCl_3 (F (1, 17) = 8.61, *p* = 0.009) and RH_Cont_PFCl_4 (F (1,17) = 7.10, *p* = 0.011). However, the AC estimates did not significantly differ between the two groups at T2 in any of the selected regions, albeit there was a slight trend in the RH_Cont_PFCl_3 (F (1,17) = 8.61, *p* = 0.02, uncorrected for multiple comparisons).

**Figure 4:**
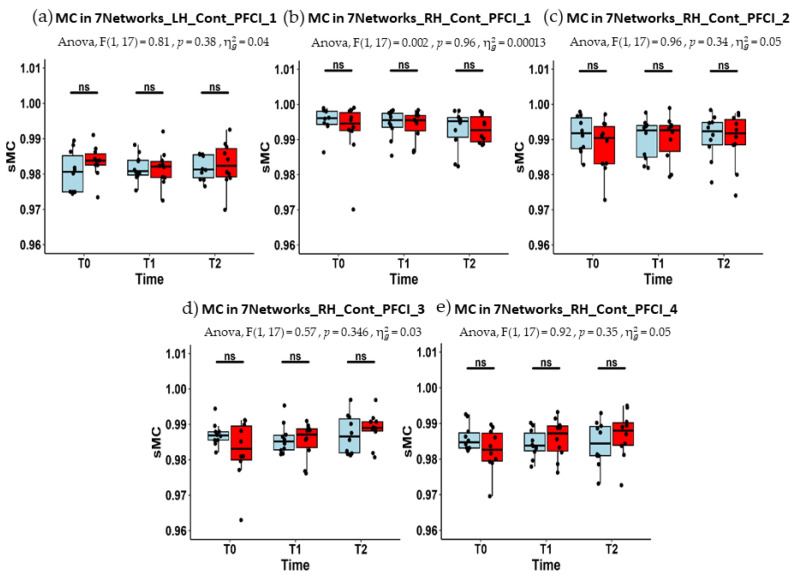
The boxplots of MC estimates (the black dots) for both group (in light blue MCI-C, in red MCI-TMS, the black points for the AC estimates of each subject) across time points (T0, T1 and T2) for the five regions, namely, LH_Cont_PFCl_1 (**a**), RH_Cont_PFCl_1 (**b**), RH_Cont_PFCl_2 (**c**), RH_Cont_PFCl_3 (**d**), and RH_Cont_PFCl_4 (**e**). In no cases, the two-way ANOVA with one within factor (observation time) and one between factors (group membership) showed MC estimates significantly affected by the interaction term, i.e., the interplay between the within and between factors. The Mann–Whitney test (U) as post-hoc test was performed but no significant difference between the groups at each time points was obtained (ns, *p* > 0.05).

In Figure 4, the boxplots of MC estimates for the selected functional brain regions, for both MCI-C and MCI-TMS groups, are shown at each time point (T0, T1, and T2). In Table 4 and Table 5, the median and the IQR of the MC estimates are reported. In none of the regions did the two-way ANOVA model result in a statistically significant group by time interaction effects. Similarly, the one-way repeated measure ANOVA model applied to MC estimates for each group separately yielded no solid evidence for significant change across time points in any of the groups. More specifically, even if the MC estimates for the RH_Cont_PFCl_3 node exhibited a slight trend towards a significant increment at T2 (vs. T0, *p* = 0.045, uncorrected for multiple comparisons) in the MCI-TMS group, the one-way ANCOVA model provided no evidence for group differences in MC changes at T1 and T2 (vs. T0).

The Spearman correlation coefficient (ρ) between the percent change in the AC estimates (at T1 and T2, with respect to T0) and the percent change in each of the three RBANS scores (namely RI story memory, line orientation, and semantic fluency) are reported, respectively, for the MCI-C and MCI-TMS groups, in Table 6 and Table 7. The percent change in the AC for the RH_Cont_PFCl_1 node exhibited a significant negative correlation with the semantic fluency variation at T1 (ρ = −0.7939, *p*-value = 0.045) for the MCI-TMS but not for the MCI-C group. Similarly, the Spearman correlation coefficient (ρ) between the percent change in the MC estimates (at T1 and T2, with respect to T0) and the percent change in each of the three RBANS scores (namely RI Story Memory, line orientation, and semantic fluency) are reported, respectively, for the MCI-C and MCI-TMS groups, in Table 8 and Table 9. In this case, the percent change in the MC for the RH_Cont_PFCl_1 node (ρ = 0.7234, *p*-value = 0.045) and for the RH_Cont_PFCl_3 (ρ = 0.7416, *p*-value = 0.045) turned out to be significantly and positively correlated with the story memory-IR at T2 for the MCI-TMS but not for the MCI-C group. No other correlations were statistically significant after FDR correction for multiple comparisons.

## 4. Discussion

This work primarily aimed at applying NCT connectome modeling to several resting-state fMRI data sets (*n* = 60) to address possible shorter (i.e., immediately after a 4-week treatment) and longer (i.e., twenty weeks after a 4-week treatment) longitudinal effects of an HF-rTMS brain stimulation on the estimated levels of average and modal functional controllability at all nodes encompassing the chosen stimulation site, i.e., the DLPFC, in a small group (*n* = 20) of MCI patients. The possible correlations between the changes in these two NCT metrics and the changes in cognitive performances at the two follow-up points of the longitudinal study were also explored and reported.

NCT connectome modeling in general, and particularly the two most widely reported NCT-derived metrics of controllability analyzed here, have been previously shown to successfully highlight the relative importance of different brain regions in modulating cognitive and behavioral performances [19,22,34]. Even if structurally-based findings comprised the intellectual premise of NCT connectome modeling in the first applications, here, in the more specific context and setting of HF-rTMS neurostimulation, this analytic framework was chosen as it might offer a different perspective for a better understanding (and possibly even a new option for effective clinical monitoring) of the HF-rTMS-induced perturbation of the functional connectome. Indeed, as the NCT model explicitly accounts for the intrinsic causal mechanism at the base of the information flow from each node throughout the functional connectome, the controllability metrics were expressly obtained to characterize any local effects at the stimulation site that could explain the neural plasticity induced by the HF-rTMS stimulation, eventually also in coupling with the changes in cognitive performances within the group of treated MCI patients. To this purpose, we first identified five regions, belonging to the PFCl control network according to a normative functional parcellation of the cerebral cortex, which anatomically encompassed the nominal target region of the stimulation (i.e., the DLPFC). Then, we estimated the level of AC and MC of these brain regions at three different time points: at baseline, i.e., immediately before (T0) the first HF-rTMS session, immediately after (T1) the 4-week HF-rTMS treatment (one session per weekday), and, finally, after 6 months (T2), i.e., twenty weeks after the end of the treatment. Thus, thanks to a double-blind experimental design, involving both real HF-rTMS and sham stimulation and three neuroimaging and neuropsychological repeated assessments, we statistically evaluated (i) with three different models the presence of any solid changes in these functional connectome metrics in response to the treatment and (ii) their possible correlations with three neurophysiological scores.

The two-way ANOVA model with one within-subject factor (observation time) and one between-subject factor (group membership) showed that the AC for the RH_Cont_PFCl_3 was differentially affected in the two groups across the three time points. Therefore, the HF-rTMS stimulation effectively caused a different modulation of the AC of the functional connectome over time, even if the median AC levels did not differ between the two groups at any specific time point, possibly due to the different initial conditions (as reflected by the observed variability in the metric at baseline). Indeed, the ANCOVA model, where the baseline levels are included as covariates, resulted in a significant change in the median AC levels of both the RH_Cont_PFCl_3 and the RH_Cont_PFCl_4 nodes, immediately after the end of all the HF-rTMS sessions (T1). More specifically, we observed that the median AC level of the MCI-TMS group was reduced significantly at this time, whereas a similar alteration was not observed in the MCI-C group. Moreover, starting from a repeated-measures ANOVA model on the AC levels of RH_Cont_PFCl_3, we further confirmed that the two groups evolved differently across times, with the MCI-TMS group exhibiting a significant reduction in the AC level of this region after 4 weeks, which also persisted after 6 months (at least relative to the initial baseline condition), and the MCI-C group exhibiting a more stable AC level. Thus, the real HF-rTMS stimulation seems to reduce effectively the capability of the RH_Cont_PFCl_3 node to drive the functional connectome towards easy-to-reach states, i.e., those states that would normally not facilitate, or possibly even hinder, the achievement of relatively better executive performances under emerging conditions of higher cognitive engagement or demand.

The same AN(C)OVA models, when applied to compare MC estimates, did not gather sufficient evidence for possible differential effects between the two groups, across all three time points. Nevertheless, when analyzing the two groups separately, for the RH_Cont_PFCl_3 node, a clear tendency towards a significant longer-term increase (at T2) was observed for the median MC level of the MCI-TMS group in comparison to the baseline condition (at T0). While the limited sample size could be a reason for the lack of statistical power in the time-by-group interaction analysis (at least after stringent FDR correction), we may still explain this partially negative result by resorting to the original interpretation of the MC metric, as usually given within the introductory literature on the adopted NCT model for the functional connectome. Namely, MC quantifies the capability of the node to efficiently drive (i.e., determine changes or switches with minimal energy expenditure) the entire connectome, facilitating brain state transitions towards “difficult-to-reach” brain states. These states would be either characterized by higher functional activations in specialized regions that normally respond to increasing task demands during cognitive engagement, such as, e.g., during working-memory task execution [20], or by higher synchronization of multiple remote regions (with respect to the stimulated node) that normally support high-order cognitive functions, such as language, with the proper combination of higher and lower activities across many distant nodes [37]. Thus, it could be even hypothesized that (more) significant MC changes would be eventually noticeable after relatively longer intervals of time from the end of the treatment, and/or if some form of re-enforcement of the HF-rTMS-induced neural plasticity occurs naturally because of daily activities, including also physical activity, which has been shown to modulate neural plasticity [38,39]. On the other hand, while not significant at the time of measurement, the observed increase in the median MC level of the MCI-TMS group after 20 weeks from the last HF-rTMS session might be anyway considered a promising result. This suggested that not only the sample size should be increased to counteract the high variability in the initial conditions among MCI patients but also the temporal positioning of the last follow-up should be ideally placed (or added) at a later stage of the clinical monitoring of the patients.

The negative correlation between the short-term variations of the AC and semantic fluency would corroborate the hypothesized (immediate) positive effect of the treatment on a brain region that is known to be crucial for this cognitive domain [40]. Similarly, in line with the current interpretation, the positive correlation between the long-term variations of the MC and story memory-IR would corroborate the hypothesized (delayed) positive effect of the treatment on the entire functional connectome when this is to be (better) controlled from the chosen stimulation site (DLPFC). Indeed, the DLPFC has a well-known (and crucial) role in the execution of complex cognitive functions [26,27], thereby the MC, which effectively quantifies the capability to drive the brain toward complex activation states, represents the more appropriate controllability metric accounting for such role. In particular, the DLPFC strongly supports the working memory functionality and the story memory-IR could be considered a highly specific RBANS score for this functionality [41]. For this reason, the long-term increment of the MC taken together with the long-term positive correlation with the variation of this cognitive score represent two elements pointing to a possible recovery of normal DLPFC functioning.

Overall, the ambivalent behavior of AC and MC effects has a clear and rational interpretation: AC measures the ability of a brain region to guide the entire system towards states that are easy to reach and would therefore occur more frequently when the subject is in a control (baseline) state of any (required or not) cognitive task [42]. In contrast, MC measures the ability of a brain region to guide the entire system towards states that are difficult to reach and would, therefore, occur more frequently only during cognitive task performances. Thus, the immediate reduction in the AC, shortly after the 4-week HF-rTMS session, together with the more delayed increase in the MC, hardly visible after 20 months from the end of the treatment, may suggest that HF-rTMS stimulation could have two different neural plasticity effects, a short-term and long-term effect: First, the stimulation reduces a possible excess of neural facilitation of “default-mode-like” (de)activation within regions that should be optimally responsive during cognitively demanding and attention-engaging tasks, such the lateral pre-frontal regions of the executive control network. Subsequently, more long-lasting neural plasticity effects may become manifest within the same region as an increase in neural facilitation of “executive-mode-like” activations. The hypothesized different direction (and timing) of these modifications would stem from the far higher number (and complexity) of the neural connections that need to be affected by the treatment and the possibility that proper training and exercise could be additionally needed after the treatment to consolidate and re-enforce these effects and make them last. The observed correlations between AC and MC changes and the RBANS scores in the MCI-TMS group would eventually corroborate this interpretation. Indeed, AC changes were negatively correlated with semantic fluency score changes at T1 (but not at T2), whereas MC changes were positively correlated with story memory-IR score changes at T2 (but not at T1) and, in both cases, for the MCI-TMS group only.

Although the proposed use of the controllability metrics seems to provide some encouragingly promising, albeit only initial, results, this work clearly represents a pilot study, and several limitations need to be clearly stated here and possibly addressed in future studies. First, the sample size is small because only 20 MCI patients successfully completed the entire study protocol, including the 4-week stimulation sessions and all three MRI and RBANS evaluations. Second, the absence of AC and MC estimates from a healthy control group undergoing (at least) the SHAM protocol, enabling the comparison of their levels between MCI and non-MCI subjects under identical conditions also represents a limiting aspect in the current interpretation of the presented data. Third, the heterogeneity of the effects of rTMS on cortical excitability (and on cognitive performances) might have certainly introduced an additional component of inter-subject variability in the AC and MC estimates in the MCI-TMS group because the stimulation site was based on standardized, not individualized, anatomical targeting [43]. All these limitations might have hindered the statistical power of the results, thus preventing the full confirmation of the observed changes in the estimated level of AC and MC across the two follow-up assessments and should be prevented in future studies.

## 5. Conclusions

In conclusion, this preliminary study revealed that controllability could represent a useful hallmark to evaluate the short- and long-term effects of an HF-rTMS treatment applied to MCI patients. Although some limitations have partially hindered the robustness of the results, the considerations about the local effects of the HF-rTMS on both AC and MC were in nice concordance with previous evidence and the typical interpretation of these metrics. This work introduced a clearly innovative aspect to advance the currently available knowledge about the effects (and consequences) of the HF-rTMS treatment for MCI. More specifically, the controllability metrics seem to account correctly for the expected role of the targeted brain region in the intrinsic mechanism of causality that is at the base of the functional switches between easy-to- and difficult-to-reach activation states. For this reason, both AC and MC might represent meaningful metrics to describe some of the possible short- and long-term neural plasticity effects that would be (expectedly) induced by any (effective) non-invasive brain stimulation, such as HF-rTMS as a candidate non-invasive intervention, not only for MCI (to prevent dementia) but also for other neurological diseases.

## Figures and Tables

**Table 1:** Between-group comparison on demographics and on the three cognitive score measures at the baseline evaluation; data are reported as median (25th, 75th percentile) or count (percentage). The statistics and the corresponding *p*-value are also reported. No statistically significant differences were detected at baseline.

Variable	MCI-TMS (*n* = 10)	MCI-C (*n* = 10)	^a^ MWU Tests ^b^ χ^2^ test	*p*-Value
Age, years	64.0 (60.8, 71.2)	70.5 (60.5, 73.8)	^a^ 93.00	0.383
Sex, male	4 (40%)	5 (50%)	^b^ 0.22	0.639
Story memory-IR at T0	15 (15, 16.5)	13 (10.8, 15)	33	0.201
Line orientation at T0	14 (11.2, 16.8)	13 (11, 16.2)	44.5	0.704
Semantic fluency at T0	13 (12, 14.5)	16 (13.5, 17)	73	0.0836

^a^ Mann–Whitney U test, MWU Tests; ^b^ Pearson’s chi-squared test, χ^2^ test.

**Table 2:** Median values and IQR of the average controllability (AC) of all selected functional ROIs across the three time points (T0, T1, and T2) for the MCI-C group are here reported. No statistically significant repeated measure ANOVA test was obtained; therefore, the AC estimates did not exhibit significant changes across time points for the MCI-C group. FDR correction for multiple comparisons was considered.

ROI Labels	Baseline—T0 Median (IQR)	After 4 Weeks—T1 Median (IQR)	After 6 Months—T2 Median (IQR)
LH_Cont_PFCl_1	1.30 (1.19–1.32)	1.26 (1.19–1.31)	1.24 (1.17–1.34)
RH_Cont_PFCl_1	1.01 (1.00–1.03)	1.03 (1.01–1.07)	1.02 (1.01–1.15)
RH_Cont_PFCl_2	1.06 (1.03–1.11)	1.07 (1.05–1.17)	1.06 (1.02–1.15)
RH_Cont_PFCl_3	1.13 (1.10–1.18)	1.16 (1.11–1.25)	1.13 (1.07–1.22)
RH_Cont_PFCl_4	1.19 (1.12–1.22)	1.21 (1.14–1.25)	1.21 (1.07–1.30)

**Table 3:** Median values and IQR of the average controllability (AC) of all selected functional ROIs across the three time points (T0, T1, and T2) for the MCI-C group are here reported. The repeated measures ANOVA test is statistically significant when considering the AC of the RH_Cont_PFCl_3 (F (17) = 4.76, *p* = 0.022). The Wilcoxon signed-rank tests for the pairwise post hoc comparison showed, after FDR correction, that in the MCI-TMS group, the AC values at T1 (*p* = 0.043) and at T2 (*p* = 0.043) significantly decreased with respect to the baseline (at T0) AC values.

ROI Labels	Baseline—T0 Median (IQR)	After 4 Weeks—T1 Median (IQR)	After 6 Months—T2 Median (IQR)
LH_Cont_PFCl_1	1.18 (1.10–1.22)	1.17 (1.14–1.25)	1.17 (1.12–1.27)
RH_Cont_PFCl_1	1.02 (1.00–1.08)	1.02 (1.00–1.03)	1.05 (1.02–1.10)
RH_Cont_PFCl_2	1.10 (1.03–1.19)	1.03 (1.02–1.15)	1.08 (1.02–1.11)
RH_Cont_PFCl_3	1.17 (1.11- 1.22)	1.07 (1.05–1.19)	1.11 (1.06–1.13)
RH_Cont_PFCl_4	1.17 (1.11–1.24)	1.10 (1.04–1.15)	1.10 (1.04–1.20)

**Table 4:** Median values and IQR of the modal controllability (MC) of all selected functional ROIs across the three time points (T0, T1, and T2) for the MCI-C group are here reported. No statistically significant repeated measure ANOVA test was obtained; therefore, the MC estimates did not exhibit significant changes across time points for the MCI-C group. FDR correction for multiple comparisons was considered.

ROI Labels	Baseline—T0 Median (IQR)	After 4 Weeks—T1 Median (IQR)	After 6 Months—T2 Median (IQR)
LH_Cont_PFCl_1	0.981 (0.975–0.985)	0.981 (0.980–0.984)	0.981 (0.979–0.985)
RH_Cont_PFCl_1	0.996 (0.994–0.998)	0.996 (0.993–0.997)	0.995 (0.991–0.996)
RH_Cont_PFCl_2	0.992 (0.987–0.996)	0.993 (0.985–0.994)	0.992 (0.988–0.995)
RH_Cont_PFCl_3	0.987 (0.986–0.988)	0.985 (0.983–0.987)	0.987 (0.982–0.992)
RH_Cont_PFCl_4	0.985 (0.983–0.987)	0.984 (0.982–0.987)	0.984 (0.981–0.989)

**Table 5:** Median values and IQR of the modal controllability (MC) of all selected functional ROIs across the three time points (T0, T1, and T2) for the MCI-TMS group are here reported. No statistically significant repeated measure ANOVA test was obtained; therefore, the MC estimates did not exhibit significant changes across time points for the MCI-TMS group. FDR correction for multiple comparisons was considered.

ROI Labels	Baseline—T0 Median (IQR)	After 4 Weeks—T1 Median (IQR)	After 6 Months—T2 Median (IQR)
LH_Cont_PFCl_1	0.984 (0.983–0.986)	0.982 (0.979–0.984)	0.982 (0.979–0.984)
RH_Cont_PFCl_1	0.995 (0.993–0.998)	0.995 (0.992–0.997)	0.993 (0.989–0.997)
RH_Cont_PFCl_2	0.990 (0.983–0.994)	0.993 (0.987–0.994)	0.992 (0.988–0.996)
RH_Cont_PFCl_3	0.983 (0.980–0.990)	0.987 (0.983–0.989)	0.989 (0.988–0.991)
RH_Cont_PFCl_4	0.983 (0.979–0.987)	0.987 (0.982–0.989)	0.988 (0.984–0.990)

**Table 6:** Spearman (ρ) correlation coefficients between the variations of AC values and of the three RBANS scores (RI-Story Memory, line orientation, and semantic fluency) at T1 and at T2 with respect to the T0 values for the MCI-C group are here presented. Statistically significant correlation values (after FDR correction) are reported in bold (*p* < 0.05). In no case did we find a statistically significant correlation between the variation of AC estimates and the variation of the neurophysiological scores for the MCI-C group.

ROI Labels And Time Point	Story Memory-IR ρ	Line Orientation ρ	Semantic Fluency ρ
LH_Cont_PFCl_1 at T1	−0.2857	0.1273	−0.0303
RH_Cont_PFCl_1 at T1	0.2917	0.4788	0.3576
RH_Cont_PFCl_2 at T1	0.3890	0.4667	0.5636
RH_Cont_PFCl_3 at T1	0.2188	0.5030	0.4788
RH_Cont_PFCl_4 at T1	0.0364	0.4303	0.3939
LH_Cont_PFCl_1 at T2	−0.0121	0.0061	−0.1951
RH_Cont_PFCl_1 at T2	−0.1763	0.3526	0.3659
RH_Cont_PFCl_2 at T2	0.1702	0.3465	0.3720
RH_Cont_PFCl_3 at T2	−0.0364	−0.0973	−0.2805
RH_Cont_PFCl_4 at T2	−0.1641	−0.0486	0.3903

**Table 7:** Spearman (ρ) correlation coefficients between the variations of AC values and of the three RBANS scores (RI-Story Memory, line orientation, and semantic fluency) at T1 and at T2 with respect to the T0 values for the MCI-TMS group are here presented. Statistically significant correlation values (after FDR correction) are reported in bold (*p* < 0.05). We found a statistically significant negative correlation between the variation in AC estimates in the RH_Cont_PFCl_1 and the variation in semantic fluency (ρ = −0.7939, *p*-value = 0.045) at T1 for the MCI-TMS group.

ROI Labels and Time Point	Story Memory-IR ρ	Line Orientation ρ	Semantic Fluency ρ
LH_Cont_PFCl_1 at T1	−0.5653	0.2364	−0.4182
RH_Cont_PFCl_1 at T1	0.3890	−0.1152	**−0.7939**
RH_Cont_PFCl_2 at T1	0.0243	−0.1879	−0.4788
RH_Cont_PFCl_3 at T1	0.0243	−0.2000	−0.2364
RH_Cont_PFCl_4 at T1	0.0851	−0.4182	−0.4424
LH_Cont_PFCl_1 at T2	0.5653	0.3161	0.1030
RH_Cont_PFCl_1 at T2	−0.5288	0.1885	−0.1636
RH_Cont_PFCl_2 at T2	−0.2067	−0.0608	0.1152
RH_Cont_PFCl_3 at T2	−0.1094	−0.0912	−0.1273
RH_Cont_PFCl_4 at T2	0.0182	−0.3100	−0.1879

**Table 8:** Spearman (ρ) correlation values between the changes in MC of the ROIs and the changes in the three RBANS scores (RI-Story Memory, line orientation, and semantic fluency) at T1 and T2 with respect to the T0 values for the MCI-C group. The statistically significant correlation values (after FDR correction) are reported in bold. In no case did we find a statistically significant correlation between the MC of the ROIs of the MCI-C patients and their three neurophysiological scores at both T1 and T2.

ROI Labels And Time point	Story Memory-IR ρ	Line Orientation ρ	Semantic Fluency ρ
LH_Cont_PFCl_1 at T1	0.7234	0.4182	0.5636
RH_Cont_PFCl_1 at T1	0.5167	−0.3576	0.3212
RH_Cont_PFCl_2 at T1	−0.3829	−0.3091	−0.5879
RH_Cont_PFCl_3 at T1	0.0668	−0.2242	−0.2606
RH_Cont_PFCl_4 at T1	0.3222	−0.1758	−0.1152
LH_Cont_PFCl_1 at T2	0.4194	0.1824	0.5000
RH_Cont_PFCl_1 at T2	0.4741	0.3222	0.0549
RH_Cont_PFCl_2 at T2	0.1823	−0.3283	−0.5366
RH_Cont_PFCl_3 at T2	0.7112	0.4620	0.6829
RH_Cont_PFCl_4 at T2	0.3282	0.2492	−0.1341

**Table 9:** Spearman (ρ) correlation values between the changes of MC of the ROIs and the changes of the three RBANS scores (RI-Story Memory, Line Orientation and Semantic Fluency) at T1 and T2 with respect to the T0 values for the MCI-TMS group. The statistically significant correlation values (after FDR correction) are reported in bold (*p* < 0.05). Statistically significant negative correlation values were found between the variation of MC and the variation RI-Story Memory of RH_Cont_PFCl_1 (ρ = 0.7234, *p*-value = 0.045) and of RH_Cont_PFCl_3 (ρ = 0.7416, *p*-value = 0.045) at T2.

ROI Labels and Time Point	Story Memory-IR ρ	Line Orientation ρ	Semantic Fluency ρ
LH_Cont_PFCl_1 at T1	0.6018	−0.2727	−0.7212
RH_Cont_PFCl_1 at T1	−0.5654	−0.2000	0.6242
RH_Cont_PFCl_2 at T1	−0.0729	−0.1030	0.5879
RH_Cont_PFCl_3 at T1	0.0304	−0.1758	0.2485
RH_Cont_PFCl_4 at T1	0.1216	0.4061	0.3333
LH_Cont_PFCl_1 at T2	−0.1520	0.0851	0.2121
RH_Cont_PFCl_1 at T2	**0.7234**	0.1824	0.3697
RH_Cont_PFCl_2 at T2	0.2736	0.4924	−0.0909
RH_Cont_PFCl_3 at T2	**0.7416**	0.4134	0.4061
RH_Cont_PFCl_4 at T2	0.5228	0.5957	0.3576

## Data Availability

All data that support the findings of this study are available upon reasonable request to the corresponding author.
